# How is place of death from cancer changing and what affects it? Analysis of cancer registration and service data

**DOI:** 10.1038/sj.bjc.6603305

**Published:** 2006-08-15

**Authors:** E Davies, K M Linklater, R H Jack, L Clark, H Møller

**Affiliations:** 1 King's College London, School of Medicine at Guy's, King's and St Thomas' Hospitals, Thames Cancer Registry, Capital House, 42 Weston Street, London SE1 3QD, UK

**Keywords:** palliative care, place of death, cancer registration, health services researches

## Abstract

We aimed to compare trends in place of cancer death with the growth of palliative care and nursing home services, and investigate demographic, disease-related and area influences on individual place of death, using registration data for 216404 patients with breast, lung, colorectal and prostate cancer and aggregate data on services in South East England. Between 1985 and 1994 there was a trend away from hospital death (67–44%), to home (17–30%) and hospice death (8–20%). After 1995, this partly reversed. By 2002, hospital death rose to 47%, home death dropped to 23%, hospice death remained stable and nursing home death rose from 3 to 8%. Numbers of palliative care services increased, but trends for hospice and nursing home deaths most clearly followed the beds available. Cancer diagnosis and treatment influenced individual place of death, but between 1998 and 2002, age and area of residence were associated with most variation. Older patients and those living in more deprived areas died more often in hospitals and less often at home. Despite more palliative care services the proportion of people dying at home has not increased. Variation by age, deprivation and area of residence is unlikely to reflect patient preference. More active surveillance and planning must support policies for choice in end of life care.

Most people say they would prefer to die at home, but in reality most patients in the UK spend their final days in hospital (WHO, 2004a). Over the last 30 years, hospices, palliative care teams and units have developed with the aim of improving care towards the end of life, and allowing people to die where they wish, if this is possible. Services have initially focused on patients with cancer, primarily because of the relative ease of predicting the course of this disease, and a national policy for supportive and palliative cancer care is now in place (House of Commons Health Committee, 2004; NICE, 2004a, 2004b). There is, therefore, increasing interest in Europe about whether data on place of death can be used as an interim measure of the success of services provided (WHO, 2004a). Figures for England and Wales revealed a trend away from death in hospital or nursing homes to hospices between 1985 and 1994, but very little change in home deaths, which remained around 26%. However, this proportion varied between regions and was lowest in South East England across all age and cancer types (Higginson *et al*, 1998). For common cancers, individual, disease-related and area of residence factors were consistently associated with, but not strongly predictive of place of death. Men, patients aged under 74 years, those with lung or colorectal cancer or living in more affluent areas were more likely to die at home than women, patients aged over 75 years, those with breast cancer or those living in less affluent areas (Higginson *et al*, 1998, 1999).

The Thames Cancer Registry covers a population of 14 million people in South East England, an area with one of the highest concentrations of hospice and palliative care services in the UK (Hospice Information, 2006). We used Registry data to describe trends in place of death for common cancers and compared these to the growth of palliative care services and nursing homes between 1985 and 2002. We then investigated the relationship between demographic, disease-related factors and individual place of death throughout the period, and the additional influence of area of residence between 1998 and 2002.

## METHODS

In the UK cancer registries record the occurrence of cancer in their residential populations as well as treatments given in the first 6 months after diagnosis. Information about death is provided by the National Health Service Central Register through the Office for National Statistics. Death certificates routinely record place of death and assign cancer as a main or contributing cause of death in part I of the certificate.

We extracted data on 216 404 residents in South East England who had been diagnosed with breast, lung, colorectal and prostate cancer between 1985 and 2002, and who died from their disease between 1985 and 2002. Cases where the only registration information was from the death certificate were not included. From death certificates, we classified death as occurring in NHS acute hospitals, hospices, long stay hospitals or nursing homes, private hospitals, at home or as unknown. We could identify nursing homes by their address, but death certificates do not distinguish deaths in hospital palliative care units from those in other wards.

We extracted data on hospice and palliative care services from Hospice Information directories for 1985–2002 (Hospice Information, 2006) and calculated the number of hospice beds, home care teams, day care services, hospital palliative care or support teams and hospital support nurses in our area. We summed home care services regardless of their funding (independent, NHS and Macmillan Cancer Relief) or base (hospices, NHS hospital or community), although there was insufficient detail about Marie Curie home services to include these. We could not deduce team size or caseload. We also obtained aggregate data on numbers of beds in registered nursing homes from the Department of Health where this was available for 1991–2001. We first plotted the proportion of deaths occurring in each of home, hospital, hospice and nursing home against the growth of different services over time. Data on acute and general hospital beds in our area were available only between 1996 and 2002 and were not plotted.

We then took death in hospice, nursing home, NHS acute hospital and at home as our four dependent variables and fitted logistic regression models to identify individual demographic, disease-related and area of residence factors predicting in turn each of these outcomes versus the others. Our first analysis for the entire period 1985–2002 included sex, age at diagnosis, whether the diagnosis was based on clinical or microscopic evidence, primary site of cancer and treatment with surgery, radiotherapy, chemotherapy or hormone therapy. We also adjusted for year of death and years since diagnosis to examine trends over time. We grouped age into four bands: <65 years, 65–74 years, 75–84 years and 85 years plus. Our second analysis explored the additional influence of area of residence for the years 1998–2002. For this, we assigned each individual to an electoral ward and a cancer network using their postcode of residence. We calculated the deprivation score for each ward using the income domain of the Indices of Multiple Deprivation (IMD) 2000 for England (Department of Environment, 2000) and assigned individuals to a quintile of deprivation ranging from most (1) to least affluent (5) wards.

We present the results of logistic regression analyses as proportions of deaths occurring in each place for each factor. Proportions are easier to interpret than odds ratios, and were derived from a back calculation from the odds ratios obtained from the logistic regression analyses. We present unadjusted and adjusted proportions to show the effect of controlling for all other factors. Our large sample size means that many small differences reach statistical significance. We draw attention only to those factors producing at least five percentage points difference – a difference which we believe a clinical service might be interested to explore further.

## RESULTS

The average age of death for patients in this cohort increased from 71.3 years in 1985 to 72.7 in 2002. The proportion dying at age of 85 years and over increased from 8 to 12% while the proportion dying between the age of 65 and 74 years dropped from 34 to 28%.

### How has place of death changed?

**Figure 1:**
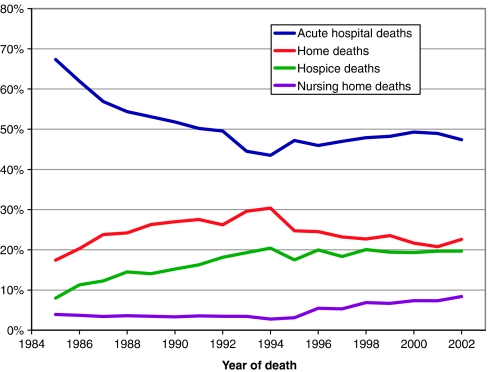
Trends for place of death for patients with breast, colorectal, lung and prostate cancer in South East England 1985–2002. *Note*: Figure excludes the proportion dying in private hospitals and patients where place of death was not known.

Figure 1 suggests that the period 1985–2002 is best considered in two phases – before and after 1994. In the first phase, hospital deaths declined from 67 to 44% – a trend that appeared to be mirrored by a combined increase in home death from 17 to 30% and in hospice death from 8 to 20%. In the second phase, however, the movement away from hospital death appeared to partly reverse. Between 1995 and 2002 hospital death rose to 47%, nursing home death to 8%, hospice death remained stable and home deaths dropped to 23%. In 2002 – the last year of the study – home death and nursing home death home appear to have increased slightly and hospital death to have decreased. During 1992 and 1995 there were changes in processing and receipt of our registry data which may be responsible for the ‘mirroring’ of trends in hospital and home deaths during this period. This artefact overlies but does not explain the reversal of overall trends which is also seen in national data for this period.

**Figure 2:**
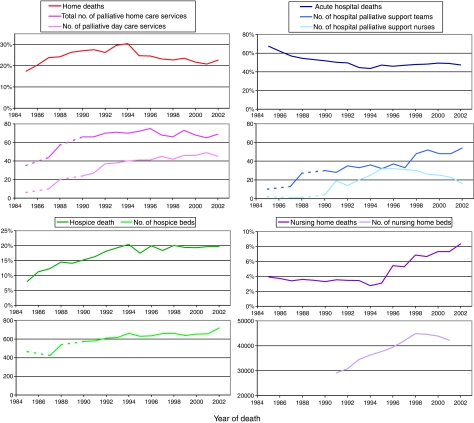
Trends in place of death for patients with breast, lung, colorectal and prostate cancer and the growth of services for care towards the end of life in South East England 1985–2002. *Note*: Department of Health Data is only available for 1991–2001 and Hospice Information Directories are not available for 1986 and 1989.

Figure 2 shows that during the first phase, while home and hospice death increased, the provision of home care services and hospice beds also increased. From 1995 onwards while nursing home death and hospital death increased, nursing home beds and, to a lesser degree, the sum of hospital palliative care services (teams and nurses) also increased. For nursing home deaths, unlike hospice deaths, there is a lag of several years between the rise of available beds in these services and deaths within them. The decline in home death occurring after 1995 did not appear to follow a substantial drop in the provision of palliative home care or day care services, which both remained stable, although during this period the availability of nursing home beds was increasing.

### Which individual and disease-related factors affect place of death?

Table 1 shows unadjusted and adjusted proportions of deaths in each place for individual demographic and disease-related factor over the entire study period. Hospital death was more likely for patients aged over 75, those with lung or breast cancer, a clinical rather than microscopic diagnosis, and those not receiving radiotherapy. Home death was more likely for those with colorectal cancer and those aged less than 75 years. Hospice death was also more likely for colorectal cancer and for those aged less than 75 years. Nursing home death increased with older age (4% for those aged 65–74 years and 12% of those aged over 85 years).

### Did place of residence affect place of death between 1998 and 2002?

Our analysis for the most recent years included area of residence as assessed by cancer network of residence and deprivation of ward of residence (Table 2). The results for demographic and disease-related factors were broadly similar to those in Table 1, although nursing home deaths become more likely for those with breast and prostate cancer. However, much more striking was the variation by area of residence. Concentrating on the nine of 13 cancer networks that we completely cover, the adjusted proportion of patients dying in hospital ranged from 39% in Sussex to 60% in West London. Home deaths ranged from 16% in Surrey, West Sussex and Hampshire to 27% in South Essex. Hospice death ranged from 10% in West London to 31% in Surrey, West Sussex and Hampshire. Nursing home deaths ranged from 4% in North London to 13% in Sussex. Of London networks, South East London had the lowest rate of hospital death (49%) and the highest rate of home death (23%). Patients from more deprived areas died more often in hospital and less often at home. There was no important deprivation gradient for nursing home or hospice death.

## DISCUSSION

### Summary of main findings

This study of 216 404 patients diagnosed and dying from four common cancers in South East England found an initial trend away from hospital death (67–44%) to home (17–30%) and hospice death (8–20%) between 1985 and 1994. After 1995 this trend partly reversed. By 2002, the proportion of hospital deaths rose to 47%, hospice deaths remained stable, home deaths dropped to 23% and nursing home deaths rose from 3 to 8%. The number and range of palliative care services increased but trends for hospice and nursing home death most clearly followed the numbers of beds available. Analysis of individual data showed that throughout the period disease-related factors had a modest influence on place of death. Patients with colorectal cancer were more likely to die at home and in hospices while patients with lung or breast cancer, no microscopic diagnosis and no radiotherapy were more likely to die in hospitals. However, between 1998 and 2002, age and place of residence were associated with most variation. Older patients were more likely to die in hospitals and nursing homes and less likely to die at home or in hospices. Patients from deprived areas were more likely to die in hospitals and less likely to die at home. There was significant variation in each place of death by cancer network of residence.

### Limitations of this study

This population-based study used data collected from medical records and death certificates for routine cancer registration. Coding officers may have missed some deaths in new nursing homes and hospices when their addresses were unfamiliar in the early part of the study period. Lack of information on death certificate on deaths in hospital palliative care units and lack of data on hospital beds meant we could not explore these trends and it is possible that excluding patients for whom we had only death certificate data from the analysis introduced some bias. Important information on patient preference for place of death, functional status, presence of a carer at home, family support, and hospital and community services received in the weeks before death (Grundy *et al*, 2004; Gomes and Higginson, 2006) is not routinely collected and is therefore missing from the individual analyses.

### Comparison to other findings

No other large UK studies have compared overall trends in place of death with the growth of services that might support patients to die in different places. However, one study of North West England between 1993 and 2000 found that proximity to a hospice or hospital increased the chances of dying there (Gatrell *et al*, 2003). Studies in the US have also found that the availability of beds and physicians affects death in hospital (Fisher *et al*, 2003a, 2003b). National bed data available for 1987 to 1994 when hospital deaths decreased showed a decline of 19% in the numbers of acute and general hospital beds (Department of Health, 2006). It, therefore, seems likely that the initial trend for increasing home death was in part driven by the decreased availability of hospital beds and the growth of hospice and palliative home care services. However, it is more difficult to explain the reversal of the trend for home death after 1995 using the routine service data that is available. The number of home care teams did not decline, hospital palliative care services were only just beginning to increase and hospital beds did not increase nationally until 2001. We can speculate that the decline in home death was due to other changes in care at home including the ability of families to provide care, the prior move of some older adults into nursing homes and the move to GP cooperatives for out of hours care. These factors could all have led to increased hospital admission and fewer home deaths.

Turning to predictors of individual place of death, our finding that younger patients, patients with colorectal cancer and those living in more affluent areas died more often at home is consistent with (Higginson *et al* 1998, 1999) analyses of a partial national registration dataset up until 1994 . However, we found that patients with breast and lung cancer were more likely to die in hospital and we were further able to show that hospital death was associated with lack of microscopic diagnosis, and no radiotherapy treatment. This suggests the late admission of patients with advanced stage of disease. Conversely our finding that a microscopically confirmed diagnosis and radiotherapy treatment were associated with home and hospice death suggests that some time within ‘the system’ may allow for referral to supportive services (Burge *et al*, 2003). A recent systematic review of factors predicting home death by Gomes and Higginson (2006) found that the six strongest predictors were patients' low functional status, their preferences, home care and its intensity, living with relatives and extended family support. Our new finding that cancer network is an important cause of variation in home death is consistent with this, and probably represents a combination of difference by area in access to home care services, and the nearness of relatives and extended family. It is very unlikely to represent underlying variation in patients' preference for place of death or functional status. Finally our finding that patients from more deprived areas were equally likely to die in hospices and nursing homes as those from affluent areas, contradicts the view that the latter may access these services more often. Inequalities in hospital and home death do, however, persist.

### Implications for practice and policy

Our findings reveal that despite increased investment in and provision of palliative care services, cancer patients in South East England remain twice as likely to die in hospital (47%) than at home (23%). The proportion dying at home is now lower than a decade ago, lower than elsewhere in the UK, and far lower than most patients would prefer. Recent national policy has set out the evidence that coordinated palliative care services can allow more people to die at home if they wish (NICE, 2004a, 2004b) and advocated equity of choice in final place of care. This study covers a period before most recent initiatives (Gold Standards Framework, 2006; Marie Curie, 2006) but the variation it finds underlines the need for much more active local surveillance to drive these policies. It also suggests that opportunities exist to learn from differing strategies, organisation and practice within cancer networks. For example, London networks might ask what it is about service provision in South East London that produces rates of home death similar to those outside London. Networks outside London might ask why hospice deaths are sometimes so high and whether nursing homes are preventing hospital admission and providing better symptom control. Our data also suggest that a good place for clinicians in primary care and acute trusts to start identifying patients in the palliative stage of disease and determining their preference for avoiding or planning admission would be the clinical diagnosis of lung or breast cancer in patients living in deprived areas for whom radiotherapy treatment is not planned. The effect of any change in practice across a network can be monitored easily by the routine work of cancer registries.

### Further research

We do not yet fully understand why place of death varies across the UK, how the nexus of factors around the patient operate together to influence this (Gomes and Higginson, 2006) and why home deaths have declined and remain so low in South East England. The imaginative use of available routine data as part of the development of cancer intelligence could help us see more clearly what is happening. For example, trends within individual cancer networks could reveal the influence of different historical patterns of service provision. Ecological studies could show us what happens when a new service such as a hospice opens locally. Mapping rates geographically by primary care trust could show the influence of services (beds and teams) and workforce (district nurses (Shipman *et al*, 2005), Marie Curie nurses and out of hours care by general practitioners). Studies of how patients move between different services and the interdependence between services are also required. Linking hospital episode statistics data with cancer registration data will, for example, allow us to explore where patients with different cancers are admitted to hospital from, how long they stay and where they are discharged to in the last months of life. The influence that admission has on rate of death in different trusts or primary care trusts explored in a similar way to US studies have done (Wennberg *et al*, 2004). Qualitative case studies of selected areas could then focus on explaining how different patterns of care are perpetuated, or how change has occurred. Finally in the context of an ageing population, changes in migration and kinship patterns we need to determine older people's preference for death in institutions, ensure that the information we have on what currently occurs is available and public so that where possible people may make their own choices in planning care towards the end of life (WHO, 2004b).

## Figures and Tables

**Table 1:** Associations of individual demographic and disease-related factors with place of death for cancer patients who died 1985–2002 in South East England

	**Acute hospital**			**Home**			**Hospice**			**Nursing home**			
	**Number**	**%**	**% Adj**	**Number**	**%**	**% Adj**	**Number**	**%**	**% Adj**	**Number**	**%**	**% Adj**	**Total**
*Age group*													
<65	28 818	47	47	17 332	28	28	12 032	20	20	956	2	2	61 670
65-74	34 777	49	47	18 104	26	26	13 045	18	20	2467	3	4	70 665
75-84	33 811	52	48	13 960	22	23	10 566	16	18	4721	7	8	64 921
85+	10 512	55	50	3717	19	22	2046	11	12	2375	12	12	19 148
													
Test for	*χ*^2^ (1 df)	574.1	51.1		1036. 6	410.7		753.7	271.2		4322.2	3206.3	
Trend	*P*	<0.001	<0.001		<0.001	<0.001		<0.001	<0.001		<0.001	<0.001	
													
*Sex*													
Male	60 626	51	51	29 552	25	25	19 793	17	17	5005	4	4	118 630
Female	47 292	48	49	23 561	24	23	17 896	18	19	5514	6	6	97 774
													
Test for	*χ*^2^ (1 df)	160.5	46.7		19.2	42.8		97.6	92.0		232.3	139.3	
Heterogeneity	*P*	<0.001	<0.001		<0.001	<0.001		<0.001	<0.001		<0.001	<0.001	
													
*Basis of diagnosis*													
Clinical	29 382	57	57	10 763	21	21	6697	13	13	3283	6	6	51 936
Microscopic	78 536	48	52	42 350	26	23	30 992	19	16	7236	4	5	164 468
													
Test for	*χ*^2^ (1 df)	1222.9	219.3		536.2	128.9		959.3	212.1		311.8	55.1	
Heterogeneity	*P*	<0.001	<0.001		<0.001	<0.001		<0.001	<0.001		<0.001	<0.001	
													
*Site*													
Colorectal	23 203	46	46	13 381	26	26	9873	19	19	2705	5	5	50 937
Lung	54 794	54	54	24 738	24	23	16 166	16	16	3575	4	4	102 071
Breast	17 375	47	56	9140	24	21	6904	18	14	2304	6	5	37 340
Prostate	12 546	48	52	5854	22	21	4746	18	18	1935	7	6	26 056
													
Test for	*χ*^2^ (3 df)	1168.1	837.7		147.8	320.6		354.4	294.2		910.8	115.9	
Heterogeneity	*P*	<0.001	<0.001		<0.001	<0.001		<0.001	<0.001		<0.001	<0.001	
													
*Had noninvestigative surgery*													
No	78 702	51	51	36 944	24	24	25 189	16	16	7527	5	5	153 570
Yes	29 216	46	54	16 169	26	22	12 500	20	17	2992	5	5	62 834
													
Test for	*χ*^2^ (1 df)	402.2	109.0		67.6	61.6		376.8	0.3		1.9	1.1	
Heterogeneity	*P*	<0.001	<0.001		<0.001	<0.001		<0.001	0.618		0.171	0.298	
													
*Had radiotherapy*													
No	79 090	52	52	34 962	23	23	24 472	16	16	8182	5	5	151 223
Yes	28 828	44	43	18 151	28	27	13 217	20	20	2337	4	5	65 181
													
Test for	*χ*^2^ (1 df)	1183.5	1240.9		548.2	329.8		528.9	425.9		323.6	2.5	
Heterogeneity	*P*	<0.001	<0.001		<0.001	<0.001		<0.001	<0.001		<0.001	0.117	
													
*Had chemotherapy*													
No	94 814	50	50	45 221	24	24	31 764	17	17	9976	5	5	187 856
Yes	13 104	46	49	7892	28	25	5 925	21	18	543	2	3	28 548
													
Test for	*χ*^2^ (1 df)	206.7	12.7		170.4	20.9		253.8	10.6		568.8	125.6	
Heterogeneity	*P*	<0.001	<0.001		<0.001	<0.001		<0.001	0.001		<0.001	<0.001	
													
*Had hormone therapy*													
No	85 818	50	50	41 640	24	24	29 243	17	17	7631	4	4	170 033
Yes	22 100	48	49	11 473	25	25	8446	18	18	2888	6	5	46 371
													
Test for	*χ*^2^ (1 df)	115.2	23.8		1.3	8.8		26.1	9.1		236.5	46.6	
Heterogeneity	*P*	<0.001	<0.001		0.263	0.003		<0.001	0.003		<0.001	<0.001	
													
*Year of death*													
1985	3278	67	67	847	17	17	388	8	8	191	4	4	4866
1986	5473	62	64	1792	20	19	996	11	10	328	4	4	8833
1987	5487	57	61	2294	24	22	1183	12	11	330	3	3	9644
1988	5976	54	59	2660	24	22	1592	14	12	396	4	3	10 989
1989	6485	53	58	3212	26	24	1718	14	12	425	3	3	12 215
1990	6558	52	57	3415	27	25	1926	15	13	420	3	3	12 659
1991	6392	50	56	3506	28	26	2074	16	14	453	4	3	12 738
1992	6274	50	55	3318	26	24	2296	18	16	440	3	3	12 660
1993	6536	45	50	4345	30	28	2834	19	17	507	3	3	14 681
1994	5342	44	50	3730	30	28	2505	20	17	339	3	2	12 279
1995	6055	47	54	3170	25	22	2242	17	15	398	3	2	12 827
1996	4917	46	53	2625	25	22	2135	20	17	583	5	5	10 704
1997	5606	47	54	2765	23	21	2188	18	15	631	5	4	11 930
1998	6463	48	55	3062	23	20	2709	20	17	927	7	6	13 501
1999	6653	48	55	3247	24	21	2675	19	17	920	7	5	13 798
2000	6849	49	56	3008	22	20	2685	19	17	1019	7	6	13 893
2001	6812	49	55	2892	21	19	2737	20	17	1019	7	6	13 919
2002	6762	47	54	3225	23	21	2806	20	17	1193	8	7	14 268
													
Test for	*χ*^2^ (1 df)	906.0	353.0		68.3	119.2		949.9	672.1		1087.3	648.8	
Trend	*P*	<0.001	<0.001		<0.001	<0.001		<0.001	<0.001		<0.001	<0.001	

Adjusted model includes: age, sex, basis of diagnosis, site, treatment (surgery, radiotherapy, chemotherapy or hormone therapy), year of death and years since diagnosis.

**Table 2:** Associations of individual demographic, disease-related and area of residence with place of death for patients who died from Breast, lung, colorectal or prostate cancer between 1998 and 2002 in South East England

	**Acute hospital**			**Home**			**Hospice**			**Nursing home**			
	**Number**	**%**	**% Adj**	**Number**	**%**	**% Adj**	**Number**	**%**	**% Adj**	**Number**	**%**	**% Adj**	**Total**
*Age group*													
<65	8756	45	45	5085	26	26	4578	23	23	411	2	2	19 521
65-74	10 018	48	46	4963	24	24	4454	21	22	1146	5	5	21 058
75-84	10 790	50	48	4180	20	21	3727	17	19	2263	11	10	21 377
85+	3975	54	50	1206	16	18	853	11	13	1258	17	14	7423
													
Test for	*χ*^2^ (1 df)	219.0	55.6		421.17	213.1		553.8	268.8		2016.8	1293.1	
Trend	*P*	<0.001	<0.001		<0.001	<0.001		<0.001	<0.001		<0.001	<0.001	
													
*Sex*													
Male	18 074	49	49	8369	23	23	7068	19	19	2305	6	6	36 629
Female	15 465	47	47	7065	22	22	6544	20	20	2773	8	10	32 750
													
Test for	*χ*^2^ (1 df)	31.2	24.0		16.3	10.0		5.2	5.7		119.7	122.8	
Heterogeneity	*P*	<0.001	<0.001		<0.001	0.002		0.023	0.017		<0.001	<0.001	
													
*Basis of diagnosis*													
Clinical	8462	56	56	2755	18	18	1977	13	13	1472	10	10	15 002
Microscopic	25 077	46	49	12 679	23	21	11 635	21	18	3606	7	9	54 377
													
Test for	*χ*^2^ (1 df)	495.0	192.4		165.9	56.3		493.1	159.9		173.2	6.5	
Heterogeneity	*P*	<0.001	<0.001		<0.001	<0.001		<0.001	<0.001		<0.001	0.011	
													
*Site*													
Colorectal	7528	45	45	3799	23	23	3564	21	21	1370	8	8	16 714
Lung	15 992	53	54	6833	23	22	5429	18	18	1460	5	5	30 306
Breast	5853	45	51	2821	22	19	2722	21	18	1184	9	10	13 026
Prostate	4166	45	44	1981	21	20	1897	20	22	1064	11	12	9333
													
Test for	*χ*^2^ (3 df)	421.7	326.2		12.1	50.3		102.9	106.7		565.1	315.6	
Heterogeneity	*P*	<0.001	<0.001		0.007	<0.001		<0.001	<0.001		<0.001	<0.001	
													
*Had noninvestigative surgery*													
No	24 850	50	50	10 836	22	22	9110	18	18	3646	7	7	49 630
Yes	8689	44	50	4598	23	22	4502	23	20	1432	7	7	19 749
													
Test for	*χ*^2^ (1 df)	208.3	1.1		17.1	0.1		176.0	12.1		0.2	0.9	
Heterogeneity	*P*	<0.001	0.300		<0.001	0.792		<0.001	0.001		0.663	0.341	
													
*Had radiotherapy*													
No	25 031	51	51	10 122	21	21	8981	18	18	3937	8	8	49 142
Yes	8508	42	41	5312	26	25	4631	23	23	1141	6	8	20 237
													
Test for	*χ*^2^ (1 df)	452.2	525.8		263.5	143.1		192.3	162.9		117.8	2.4	
Heterogeneity	*P*	<0.001	<0.001		<0.001	<0. 001		<0.001	<0.001		<0.001	0.122	
													
*Had chemotherapy*													
No	27 748	49	49	11 977	21	21	10 409	19	19	4764	8	8	56 216
Yes	5791	44	47	3457	26	23	3203	24	20	314	2	5	13 163
													
Test for	*χ*^2^ (1 df)	122.7	25.4		150.9	25.9		227.3	24.1		508.2	97.0	
Heterogeneity	*P*	<0.001	<0.001		<0.001	<0.001		<0.001	<0.001		<0.001	<0.001	
													
*Had hormone therapy*													
No	27 739	50	50	12 320	22	22	10 839	19	19	3655	7	7	55 958
Yes	5800	43	46	3114	23	25	2773	21	20	1423	11	7	13 421
													
Test for	*χ*^2^ (1 df)	174.6	28.8		8.8	21.9		11.5	2.3		259.4	8.9	
Heterogeneity	*P*	<0.001	<0.001		0.003	<0.001		0.001	0.130		<0.001	0.003	
													
*Network of residence*													
North East London	3946	56	56	1353	19	19	1346	19	19	347	5	5	7080
North London	3314	54	55	1224	20	19	1173	19	20	261	4	4	6128
South East London	3533	49	49	1697	24	23	1549	22	22	328	5	5	7204
South West London	2899	49	51	1111	19	17	1472	25	27	313	5	5	5886
West London	3988	58	60	1391	20	19	693	10	10	417	6	6	6829
TCR part of Mount Vernon	2459	50	53	1415	29	26	488	10	11	458	9	9	4924
South Essex	2121	52	54	1165	29	27	415	10	11	338	8	8	4067
Kent & Medway	3663	42	42	2139	24	23	2186	25	26	688	8	7	8793
Surrey, West Sussex and Hampshire	2688	43	48	1232	20	16	1771	28	31	349	6	5	6262
Sussex	2642	38	39	1515	22	20	1625	23	25	1046	15	13	7001
TCR part of Central South Coast	327	25	25	249	19	18	367	28	32	150	12	10	1301
TCR part of Mid Anglia	1734	52	53	791	24	22	473	14	15	328	10	9	3360
TCR part of West Anglia	201	41	45	140	28	24	48	10	11	48	10	9	492
Other/NK	24	46	55	12	23	21	6	12	6	7	13	12	52
													
Test for	*χ*^2^ (12 df)	1361.2	1219.7		399.9	401.8		1613.5	1687.6		943.1	690.8	
Heterogeneity	*P*	<0.001	<0.001		<0.001	<0.001		<0.001	<0.001		<0.001	<0.001	
													
*IMD*													
Most affluent 1	5020	42	42	2926	25	25	2458	21	21	896	8	8	11 850
2	5965	46	45	3106	24	23	2449	19	21	1125	9	8	13 071
3	6754	48	47	3177	23	22	2620	19	22	1099	8	7	13 936
4	7692	51	48	3235	21	20	2891	19	24	1123	7	7	15 181
Least affluent 5	8105	53	49	2988	19	18	3190	21	25	834	5	7	15 330
NK	3	27	23	2	18	17	4	36	72	1	9	5	11
													
Test for	*χ*^2^ (1 df)	362.7	92.2		132.4	152.0		0.8	58.9		67.5	4.5	
Trend	*P*	<0.001	<0.001		<0.001	<0.001		0.387	<0.001		<0.001	0.035	

Adjusted model includes: age, sex, basis of diagnosis, site, treatment (surgery, radiotherapy, chemotherapy or hormone therapy), cancer network of residence and deprivation.
